# Whey Protein Hydrogels and Emulsion Gels with Anthocyanins and/or Goji Oil: Formation, Characterization and In Vitro Digestion Behavior

**DOI:** 10.3390/antiox14010060

**Published:** 2025-01-07

**Authors:** Abdullah S. Seddiek, Kaiwen Chen, Fanlin Zhou, Muhindo Mwizerwa Esther, Abdelaziz Elbarbary, Hazem Golshany, Angelo Uriho, Li Liang

**Affiliations:** 1 State Key Laboratory of Food Science and Resources, Jiangnan University, Wuxi 214122, China; abdullahsaad@alexu.edu.eg (A.S.S.); abdelaziz.elbarbary@fagr.bu.edu.eg (A.E.); hazemgolshany@cu.edu.eg (H.G.);; 2 School of Food Science and Technology, Jiangnan University, Wuxi 214122, China; 3 Department of Food Science, Faculty of Agriculture (Saba Basha), Alexandria University, Alexandria 21531, Egypt; 4 Dairy Science Department, Faculty of Agriculture, Benha University, Moshtohor 13736, Egypt; 5 Food Science Department, Faculty of Agriculture, Cairo University, Giza 12613, Egypt

**Keywords:** anthocyanins, goji oil, emulsion gel, spatial partition, digestion

## Abstract

Whey protein isolate (WPI) has functional properties such as gelation and emulsification. Emulsion gels combine the benefits of both emulsions and hydrogels. In this study, WPI hydrogels and emulsion gels were developed with goji oil (GO) as the oil phase by the inclusion of blueberry extract (BE) in the protein matrix. Heat-denatured WPI (hWPI) particles and emulsions were characterized in terms of size distribution, ζ-potential, interfacial protein, and anthocyanin partition. The inclusion of anthocyanins-rich blueberry extract led to the aggregation of hWPI particles, but it also increased the interfacial protein of 10% goji oil emulsions to 20% and decreased their size distribution to 120 and 325 nm. WPI hydrogels and emulsion gels were analyzed in terms of their water-holding capacity, which decreased from 98% to 82% with the addition of blueberry extract and goji oil. Syneresis, rheological, and morphological characteristics were also analyzed. The gelation time of hWPI particles and emulsions was shortened from 24 h to 12 h when incorporating blueberry extract to form a dense network. The network was the most homogeneous and densest in the presence of 3% blueberry extract and 5% goji oil. The co-inclusion of blueberry extract and goji oil increased the syneresis during the freeze–thaw cycles, with the values rising from 13% to 36% for 5% BE hydrogel and BE-containing emulsion gels after the first cycle. All WPI hydrogels and emulsion gels exhibit predominantly elastic behavior. Moreover, anthocyanin release, antioxidant activity, and the fatty acid composition profile were also analyzed during in vitro digestion. Soluble and free anthocyanins in the digested medium were reduced with the goji oil content but increased with the blueberry extract content. The stability of polyunsaturated fatty acids in the digested medium was improved by the addition of blueberry extract. The antioxidant activity of the digested medium increased with the content of blueberry extract but decreased with the content of goji oil. The ABTS^∙+^ scavenging capacities decreased from 63% to 49% by increasing the content of GO from 0% to 10% and they increased from 48% to 57% for 5% BE and 10% GO emulsion gels as the BE content increased from 0% to 5% after 6 h of digestion. The data gathered should provide valuable insights for future efforts to co-encapsulate hydrophilic and hydrophobic agents, thereby enhancing their stability, bioavailability, and functional properties for potential applications in food industries.

## 1. Introduction

Whey proteins have functional properties such as gelation and emulsification. Cold-set hydrogels can be formed by the heat-induced denaturation of whey proteins, followed by cross-linking with salts. Calcium ions may shield electrostatic repulsion or bind specifically to proteins to reduce charge density. Moreover, calcium ions may also act as salt bridges between negatively charged groups on neighboring protein molecules [1]. The gelation of whey proteins can also be induced by bioactive components (e.g., ascorbic acid and gallic acid) as gelling agents or accessories [2,3]. Owing to acidification or interaction with proteins of bioactive components, their inclusion may affect the gelation of proteins and their gel texture. Emulsion gels consist of oil droplets trapped in a gel network and combine the benefits of both emulsions and hydrogels. The microstructure and physicochemical properties of hydrogels and emulsion gels can be tailored by modulating the fraction of the oil phase and the presence of bioactive components [3,4].

Anthocyanins (ACNs) are naturally occurring glycosylated anthocyanidins. These constitute the most important group of water-soluble plant colorants. Anthocyanins have antioxidant and anti-inflammatory activities, and their intake is associated with a low incidence of chronic and degenerative diseases [5,6,7]. The use of anthocyanins in food and pharmaceutical products is hindered by their sensitivity to environmental factors such as pH, temperature, and oxygen [5,8,9]. The encapsulation of bilberry anthocyanins was performed by forming a W/O emulsion, followed by thermal gelation of the inner aqueous phase of anthocyanins and whey protein [10]. In addition, O/W emulsions with bilberry seed oil were stabilized by a GDL-induced gel of anthocyanins and whey protein, where anthocyanins showed a protective effect against lipid oxidation [11]. It has been reported that anthocyanins have no effect on the gelation process and final gel strength of the GDL-induced gel of whey protein [12]. However, when anthocyanin-rich extract was obtained from the black soybean seed coat, the extract inclusion decreased the soy protein aggregates and produced more string-of-beads structures in the GDL-induced gel network compared with the control [13].

Protein-based emulsion gels contain oil droplets and protein networks, which provide different micro-environments for encapsulating and delivering bioactive compounds [14,15]. Soy proteins and anthocyanins were covalently bonded to form nanoparticles, which were used to prepare Pickering emulsions with extraordinary emulsification stability, improved oxidative stability, and resistance to simulated digestion [16]. Protein–polyphenol complexes have been widely used to stabilize O/W emulsions through non-covalent interactions [17,18]. The protein layer undergoes reversible collapse when the amount of protein exceeds the maximum molecule density, and the reform of the interfacial membrane is driven by the attraction force of protein at the oil–water interface [19]. The adsorption of proteins on the surface of oil droplets and the texture of emulsion gels were also dependent on oil type [20,21].

Goji oil (GO), derived from goji berries, is rich in unsaturated fatty acids and carotenoids. In this study, hydrogels of whey protein isolate (WPI) and anthocyanins-rich blueberry extract (BE), and their emulsion gels with GO, were prepared with calcium as a cross-linker. The effects of varying the BE and GO contents on both WPI aqueous solutions and emulsions were investigated in terms of size distribution, ζ-potential, interfacial protein, and the spatial partition of anthocyanins. Additionally, hydrogels and emulsion gels were characterized in terms of their water-holding capacity, syneresis, rheological and morphological characteristics, as well as in vitro digestion. Anthocyanin quantitation, fatty acid composition profile, and antioxidant activity were analyzed in the digested hydrogels and emulsion gels. This study aimed to provide valuable insights for future efforts to co-encapsulate hydrophilic and hydrophobic agents, thereby enhancing their stability and nutritional properties for potential applications in the food and pharmaceutical industries.

## 2. Materials and Methods

### 2.1. Materials

Whey protein isolate (WPI, ~92%, Biopro) was procured from Davisco International Inc. (Le Sueur, MN, USA). A blueberry extract (BE) containing 25% anthocyanin was sourced from Baoji Runmu Agricultural Development Co., Ltd. (Shaanxi, China). Goji oil (Marque Sealong) was obtained from Shanxi Sealang Biological Chemical Co., Ltd. (Shaanxi, China). Cyanidin-3-O-glucoside chloride (Cyd-3-O-GluCl, HPLC grade, ≥95%) was acquired from Shanghai Yuanye Bio-Technology Co., Ltd. (Shanghai, China). Pepsin (extracted from porcine gastric mucosa, ≥500 U/mg) and pancreatin (extracted from porcine pancreas, 4 × USP specifications) were purchased from Sigma-Aldrich Chemical Co. (St. Louis, MO, USA). Formic acid (HPLC grade, ≥98%), acetonitrile (HPLC grade, ≥99.9%), calcium chloride, and other analytical-grade reagents were obtained from Sinopharm Chemical Reagent Co., Ltd. (Shanghai, China).

### 2.2. Samples Preparation

WPI at 6.5% (*w*/*w*) was dissolved in deionized water, heated at 85 °C for 30 min, and then cooled to room temperature to produce heat-denatured WPI (hWPI) [22]. BEs at 0%, 3%, and 5% were incorporated into hWPI aqueous solutions under stirring. The solutions of hWPI without and with BE were mixed with 0%, 5%, and 10% GO with a high-speed blender at 16,971× *g* for 5 min. The mixture was then degassed under a vacuum and subsequently passed twice within an AH-2010 high-pressure homogenizer (ATS Engineering Ltd., Toronto, ON, Canada) at 500 bar and 10 °C. Calcium chloride (0.2 M) was added to induce the gelation of WPI and BE mixtures and their stabilized GO emulsions with a final salt concentration of 10 mM using a magnetic stirrer.

### 2.3. Characterization of WPI Aqueous Solutions and Emulsions

#### 2.3.1. Size Distribution and ζ-Potential

Samples were diluted 500-fold with deionized water. Size distribution and ζ-potential were, respectively, characterized using dynamic light scattering (DLS) and phase-analysis light scattering (PALS) at 25 °C using a NanoBrook Omni Analyzer (Brookhaven Instruments Ltd., New York, NY, USA) equipped with a He/Ne laser (λ = 633 nm), following the method described by Khan et al. [23].

#### 2.3.2. Determination of Interfacial Protein Percentage

Emulsions were centrifuged at 30,000× *g* for 40 min using a CP100NX ultracentrifuge (Koki Holding Co., Ltd., Hitachi Naka, Ibaraki, Japan). The protein content in the aqueous phase of the emulsions is the sum of WPI in the subnatant and the sediment. The protein contents in the whole emulsion and the aqueous phase were quantified using the Kjeldahl method [20]. The IPP was calculated using the following formula:

(1) $$Interfacial\;protein\;percentage\;(\%)=\frac{\;\mathrm{WPI}\;\mathrm{in}\;\mathrm{the}\;\mathrm{emulsion}-WPI\;in\;the\;aqueous\;phase}{WPI\;in\;the\;emulsion}\times 100$$

#### 2.3.3. Infrared Spectroscopy

Infrared (IR) spectroscopic analysis was performed using a Thermo Nicolet FTIR iS10 spectrometer (Thermo Fisher Scientific Co., Ltd., Waltham, MA, USA) equipped with an attenuated total reflectance (ATR) module. Sample characterization was conducted by direct application onto the ATR crystal surface under ambient conditions. The spectra in the mid-infrared region (4000–800 cm⁻^1^) were collected at 4 cm⁻^1^ resolution and 32 scans [24].

#### 2.3.4. Encapsulation Efficiency and Spatial Partitioning of Anthocyanin

The spatial partitioning of anthocyanin in samples was determined by following the methodology described by Chen et al. [20]. Emulsions were centrifuged at 15,000× *g* for 1 h at 4 °C three times. The anthocyanin content in the aqueous phase of the emulsions (*AC_a_*) is the sum of anthocyanin in both the subnatant and the sediment. After the subnatant was adjusted to the isoelectric point of whey protein at pH 5.1 and then centrifuged at 15,000× *g* for 1 h at 4 °C, free anthocyanin (*AC_f_*) was in the supernatant. The amount of anthocyanin added initially to the emulsions was recorded as *AC_t_*. The spatial partitioning of anthocyanin in the emulsions was determined as follows:

(2) $$Anthocyanin\;in\;the\;aqueous\;phase\;(\%)=\frac{{\;AC}_{a\;}\;}{{AC}_{t}\;}\times 100$$

(3) $$Free\;anthocyanin\;in\;the\;aqueous\;phase\;(\%)=\frac{{\;AC}_{f\;}\;}{{AC}_{t}\;}\times 100$$

(4) $$Anthocyanin\;at\;the\;interface\;(\%)=\frac{{\;AC}_{t\;}-{AC}_{a\;}\;}{{AC}_{t}\;}\times 100$$

(5) $$\mathrm{WPI-encapsulated}\;anthocyanin\;in\;the\;aqueous\;phase\;(\%)=\frac{{AC}_{a}-{AC}_{f}}{{AC}_{t}}\;\times \;100$$

(6) $$Totally\;encapsulated\;anthocyanin\;(\%)=\frac{{AC}_{t}-{AC}_{f}}{{AC}_{t}}\;\times \;100$$

#### 2.3.5. Quantification of Anthocyanin Using HPLC

The quantification of anthocyanin was performed using a Waters Alliance high-performance liquid chromatography (HPLC) system equipped with an e2695 separation module and a 2998 photo-diode array (PDA) detector (Waters Corporation, Milford, OH, USA). Each sample (50 μL) was injected into a Symmetry C8 column (4.6 × 250 mm, 5 μm, Waters, Milford, Massachusetts, USA) maintained at 35 °C. The mobile phase consisted of formic acid/Milli-Q water (1:99, *v*/*v*, as solvent A) and acetonitrile (as solvent B). Prior to injection, the mobile phases were degassed by shaking and sonication to remove air bubbles. Detection was carried out using scanning wavelengths between 200 and 600 nm, with a specific observation at 520 nm. The gradient elution program was as follows: 0–3 min, 6% B; 3–6 min, 6–13% B; 6–26 min, 13–30% B; 26–28 min, 30–95% B; 28–32 min, 95–6% B; 32–35 min, 6% B. The flow rate was maintained at 1.0 mL/min, and each sample had a total run time of 35 min [25].

To establish the standard curve, 5 mg of Cyd-3-O-GluCl was dissolved in 10 mL of 0.1% formic acid aqueous solution to prepare a stock solution of 500 mg/L. This stock solution was subsequently diluted to prepare eight different concentrations (100, 50, 25, 5, 2, 1, 0.5, and 0.1 mg/L) for calibration. The samples were filtered through a 0.22 μm SCRC syringe filter before HPLC analysis. The absorbance was measured using scanning wavelengths between 200 and 600 nm, with a specific wavelength at 520 nm (Figures S1 and S2). The calibration curve was determined to be as follows:

*Y =* 119330*X* − 43648 (*R*^2^
*=* 0.9999) (7)

where *Y* represents the absorbance units and *X* is the concentration in mg Cyd-3-O-GluCl equivalent/L, with *R*^2^ indicating the correlation coefficient.

### 2.4. Characterization of Hydrogels and Emulsion Gels

#### 2.4.1. Water-Holding Capacity

The water-holding capacity (WHC) of hydrogels and emulsion gels was measured using the centrifugal method [26]. Specifically, 3 g of the gels in a 50 mL centrifuge tube was centrifuged at 12,000× *g* at 4 °C for 20 min. After centrifugation, the excess water was carefully drained, and the sample surface was meticulously dried with filter paper. The centrifuge tube containing the sample was accurately weighed both before and after centrifugation. The gel’s WHC was calculated using the following formula:

(8) $$WHC\;(\%)=\frac{W_{t}-W_{r}}{W_{t}}\;\times \;100$$

where *W_t_* is the total water weight in the gels, and *W_r_* is the weight of water released. Each sample was evaluated a minimum of three times.

#### 2.4.2. Syneresis (%) by Freeze–Thaw Cycles

The freeze–thaw stability of the samples was assessed by measuring the syneresis (%) following multiple freeze–thaw cycles according to the method of Liang et al. [26]. Specifically, 3 g of gels was placed in a 50 mL centrifuge tube and centrifuged at 1000× *g* for 8 min at 25 °C. The expelled water was removed, and the initial weight of the gels was recorded. The tubes were then frozen at −20 °C for 24 h and subsequently thawed at 30 °C for 2 h in a water bath. After thawing, the tubes were centrifuged again to remove water and re-weighed. This freeze–thaw cycle was repeated up to five times. The syneresis (%), indicating the amount of water lost from the gel after freeze–thaw treatment, was calculated using the following equation:

(9) $$\mathrm{Syneresis}\;(\%)=\frac{W_{i}-W_{c}}{W_{i}}\;\times \;100$$

where *W_i_* represents the initial weight of the gel before the 1st or nth freeze–thaw cycle, and *W_c_* represents the weight of the gel after the nth cycle of freeze–thawing.

#### 2.4.3. Rheological Characteristics

The rheological properties of the gels were evaluated using a shear rheometer MCR-302 (Anton Paar, Graz, Austria). An aluminum plate with a diameter of 50 mm was used, and the gap between the plate and the sample was set to 1000 μm. The sample was allowed to stabilize on the measurement cell for 180 s at 25 °C before initiating the measurement. The shear viscosity was determined by varying the shear rate from 0.1 to 10 s^−1^. Additionally, the storage modulus (G′) and loss modulus (G″) were measured over a frequency range of 0.1 to 10 rad/s while maintaining a constant strain of 1%.

#### 2.4.4. Morphological Characteristics

The microstructure was examined using a confocal laser scanning microscope (CLSM, LSM 710, Zeiss AG, Jena, Germany). Nile red (0.2% *w*/*v* in methanol) was used as a fluorescent dye for the oil phase, and Nile blue A (0.2% *w*/*v* in Milli-Q water) was used as a fluorescent dye for the protein. The dyes were mixed in a 1:1 ratio for approximately 5 min. To visualize the microstructure of hydrogels or emulsion gels, 100 μL of the dye mixture was added to 200 μL of the sample, followed by the addition of CaCl_2_ to achieve a final salt concentration of 10 mM. A 10 μL aliquot of samples mixed with fluorescent dyes was then placed on a glass slide. The CLSM images were captured using a 40× magnification lens with a He-Ne laser. Nile red and Nile blue A were excited at wavelengths of 543 nm and 633 nm, respectively.

### 2.5. In Vitro Digestion of Hydrogels and Emulsion Gels

The in vitro gastrointestinal (GI) digestion of the gels was conducted following the standards and methodologies outlined in the United States Pharmacopeia [27], employing a two-step GI model that simulates the stomach and small intestine phases, according to the method of Svelander et al. [28] and Remondetto et al. [29]. In a 50 mL screw-capped glass tube, 3 g of gels was combined with 9 mL of simulated gastric fluid (SGF) at pH 1.2 (~84 mM HCl), containing 0.32% (*w*/*v*) pepsin and 0.2% (*w*/*v*) NaCl at 37 (±0.5) °C under stirring at 100 rpm for 2 h. Subsequently, the pH was adjusted to 7.4 using 0.2 N NaOH, and 1% (*w*/*v*) pancreatin in 50 mM of potassium phosphate buffer (pH 7.4) was added. The samples were incubated in the simulated intestinal phase for an additional 4 h. The samples were collected at regular intervals in triplicate for analysis.

#### 2.5.1. Quantitation of Anthocyanin

The collected dissolution media were centrifuged at 8000× *g* for 10 min at 4 °C, and the soluble anthocyanin in the supernatant was quantified using HPLC. Additionally, the pH of the digestion solution was adjusted to the pI of hWPI and centrifuged at 15,000× *g* for 20 min at 4 °C, and the free anthocyanin in the supernatant was quantified using HPLC [20].

#### 2.5.2. Antioxidant Activity

The antioxidant activity of the digested samples was assessed by using the ABTS [2,2′-Azino-bis(3-ethylbenzothiazoline-6-sulfonic acid)] assay [30]. In brief, 4.9 mM of potassium persulfate was mixed with 7 mM of ABTS aqueous solution in the dark for 12 h to generate ABTS^∙+^ radicals. The ABTS^∙+^ solution was diluted with 50 mM of phosphate buffer (pH 7.4) to achieve an absorbance of 0.70 (±0.02) at 734 nm. After a 2.5 μL sample was mixed with 1 mL of the diluted ABTS^∙+^ solution, the absorbance was measured at 734 nm using a Synergy H1 microplate reader (Agilent Co., Ltd., New York, NY, USA). The ABTS^∙+^ scavenging capacity was calculated using the following formula:

(10) $${ABTS}^{∙+}\;scavenging\;capacity\;(\%)=\frac{A_{C}-A_{S\;}}{A_{C}}\times 100$$

where *A_C_* and *A_S_* are, respectively, the absorbance of the control reaction and the test sample.

#### 2.5.3. Extraction of Goji Oil

The extraction of GO from the digested samples was performed using a chloroform/methanol extraction method, as described by Folch et al. [31], with minor modifications. Briefly, the entire sample was mixed with 50 mL of chloroform/methanol (2:1, *v*/*v*) for 4 min. The resulting extract was then combined and equilibrated with ¼ volume of NaCl solution (0.86%, *w*/*w*) and transferred to a separation funnel to achieve phase separation. The chloroform layer was collected and evaporated under reduced pressure using a rotary evaporator.

#### 2.5.4. Fatty Acid Composition Profile of Digested and Non-Digested Goji Oil

The composition of fatty acid methyl esters was analyzed using gas chromatography, as described by Bakry et al. [32]. Exactly 50 mg of GO was dissolved in 1 mL of hexane and 500 μL of 2 M KOH–methanol under stirring for 4 min. Following the shaking process, the upper layer was collected, dried using Na_2_SO_4_, and filtered through a 0.22 μm syringe filter. The analysis was performed using a gas chromatograph (Agilent 7820A) equipped with a capillary column (TRACE TR-FAME, 60 m × 0.25 mm × 0.25 μm, Thermo Fisher, Waltham, MA, USA). The temperature gradient program started at 60 °C for 3 min, then increased to 175 °C at a rate of 5 °C/min for 15 min, and further increased to 220 °C at a rate of 2 °C/min for 20 min. The split ratio was set to 1:100, and the injector and detector temperatures were maintained at 250 °C. Nitrogen was used as the carrier gas at a flow rate of 1.2 mL/min.

### 2.6. Statistical Analysis

All samples were prepared and analyzed at least in triplicate. Data are presented as mean values ± standard deviations. Significant differences (*p* < 0.05) were determined using a one-way analysis of variance (ANOVA, Duncan’s test) conducted with IBM SPSS Statistics for Windows, version 25.0 (IBM Corp., Armonk, New York, NY, USA).

## 3. Results

### 3.1. Characterization of WPI Aqueous Solutions and Emulsions

#### 3.1.1. Size Distribution and ζ-Potential

**Figure 1:**
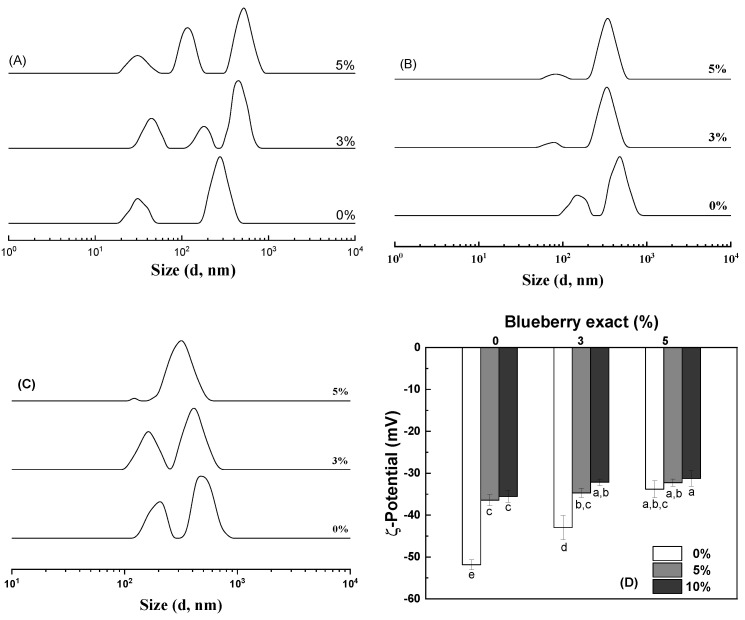
Size distribution (**A**–**C**) and ζ-potential (**D**) of WPI particles (**A**) and emulsions with 5% (**B**) and 10% (**C**) goji oil in the presence of 0%, 3%, and 5% blueberry extract. Values with different letters are statistically significantly different (*p* < 0.05).

Figure 1A illustrates that the hWPI particles exhibited a bimodal size distribution of approximately 30 and 265 nm. The addition of BE at 3% and 5% transitioned the size distribution of hWPI from bimodal to trimodal, resulting in the appearance of larger size distributions of approximately 435 and 530 nm, respectively. At the same time, the ζ-potential values of the hWPI particles decreased from −52 mV to −34 mV as the BE content increased from 0% to 5% (Figure 1D). This phenomenon may be attributed to the weakly acidic nature of anthocyanins and other constituents of BE, which dissociate and release protons into the solution, thereby lowering the pH (Table S1) and reducing the charge density on the particle surface [33]. Anthocyanin is a major component in blueberry extract. Anthocyanins can interact with whey proteins mainly through hydrophobic interactions. Moreover, electrostatic interactions, hydrogen bonding, and van der Waals interactions have also been reported between anthocyanins and whey proteins. The thermal denaturation of whey proteins improves their interaction with anthocyanins [34,35,36]. Therefore, it was suggested that the interaction between hWPI and BE leads to the formation of large molecular aggregates. This aggregation occurs through a process known as co-pigmentation, where each hWPI molecule associates with approximately 0.5 to 0.7 anthocyanin molecules from the BE. Co-pigmentation is facilitated by the unfolding of WPI during heat treatment, which exposes functional groups previously buried within the tertiary structure of the protein. These newly exposed groups, particularly the carbonyl moieties of the peptide bonds, can form hydrogen bonds with the phenolic hydroxyl groups of anthocyanins. This specific molecular interaction not only drives the formation of larger complexes but also significantly enhances the stability of anthocyanin molecules. The resulting hWPI–anthocyanin complexes exhibited improved resistance to degradation compared to free anthocyanins, potentially leading to enhanced color stability and increased bioactivity retention in food systems [37,38].

The hWPI-stabilized emulsions with 5% GO had two size distributions of 150 and 480 nm, which decreased to approximately 80 and 340 nm, respectively, upon the addition of 3% and 5% BE (Figure 1B). The hWPI-stabilized emulsions with 10% GO had two size distributions around 205 and 480 nm, which also decreased in the presence of BE (Figure 1C). The 10% GO emulsions had two size distributions around 120 and 325 nm when the BE content was 5%. At the same time, the fraction of the small size distribution decreased, and the fraction of the large size distribution increased with increasing BE content. A small size distribution has been reported for lipid-free hWPI aggregates and a large size distribution for hWPI-stabilized oil droplets [39,40]. The size distribution of GO emulsions was more homogeneous than that of lipid-free particles, possibly because the oil layer likely minimized the short-range van der Waals attractive forces among the particles but enhanced the electrostatic repulsive forces due to the charge disparity between the oil and water phases [41]. The emulsions with 5% and 10% GO had ζ-potential values of approximately −36 mV, which were lower than those of the hWPI particles (Figure 1D). The ζ-potential of the GO emulsions decreased to −32 mV as the BE content increased to 5% (Figure 1D). There is a positive correlation between the ζ-potential and particle stability [42]. All hWPI particles and emulsions had ζ-potential absolute values above 30 mV (Figure 1D), suggesting that electrostatic repulsion and steric stabilization were sufficient to stabilize them in aqueous solution [43,44].

#### 3.1.2. Interfacial Protein Percentage

**Figure 2:**
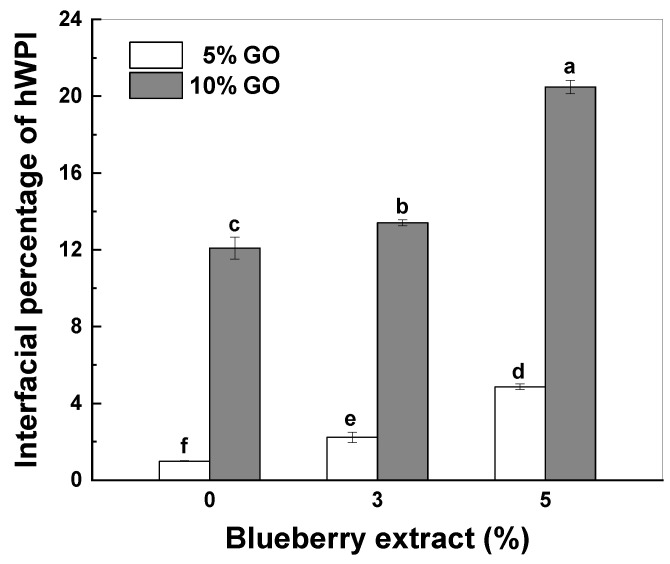
Interfacial protein percentage and content in goji oil (GO) emulsions stabilized by heat-denatured WPI in the absence and presence of blueberry extract. Values with different letters are statistically significantly different (*p* < 0.05).

Proteins are partitioned in the aqueous phase and at the oil–water interface in emulsions. A positive correlation has been established between the interfacial protein and the physical stability of the emulsions [24]. Figure 2 illustrates that the interfacial percentage of hWPI increased with increasing GO and BE content. When the GO content was 5%, the interfacial percentage of hWPI increased from 0.99% to 4.86% as the BE content increased from 0% to 5%. When the GO content was 10%, the interfacial percentage of hWPI increased from 12.09% to 20.48% as the BE content increased from 0% to 5%. When protein molecules carry a high net surface charge, a relatively high energy barrier must be overcome for adsorption [45]. The inclusion of BE reduced the surface charges of the hWPI particles (Figure 1D), improving protein adsorption on the surface of the GO droplets (Figure 1 and Figure 2). Therefore, the size of the oil droplets decreased as the BE content increased when the GO content was 10% (Figure 1C).

#### 3.1.3. FTIR Spectral Analysis

**Figure 3:**
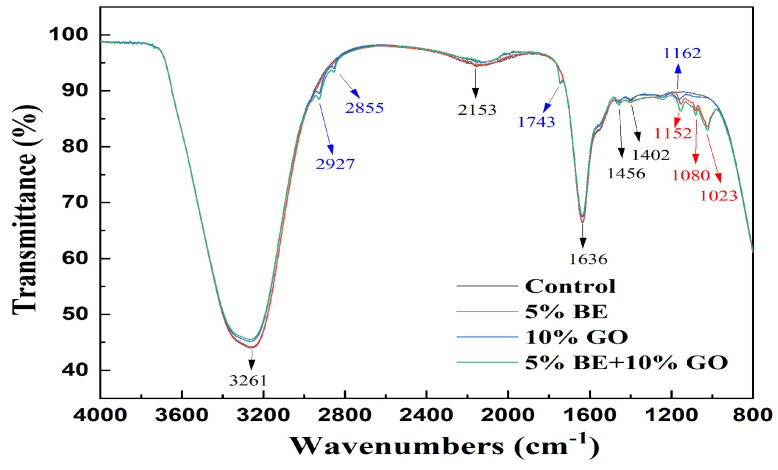
FTIR spectra of WPI particles and emulsions with and without different contents of blueberry extract (BE) and goji oil (GO).

IR spectra can be used to reveal intermolecular interactions and structural modifications. Figure 3 shows that the control sample exhibited characteristic protein bands. A broad absorption band of hWPI particles around 3262 cm^−1^ is attributed to O-H stretching. The O-H stretching band shifted to 3258 cm^−1^ in the presence of 5% BE, suggesting hydrogen bonding between hWPI and anthocyanins. The O-H stretching band shifted to 3269 cm^−1^ upon forming the emulsions of 10% GO, which was independent of the presence of BE.

The amide I region of hWPI particles around 1636 cm^−1^ primarily originates from C=O stretching vibrations and directly relates to the protein backbone conformation, the amide II band around 1457 cm^−1^ is attributed to N-H stretching vibrations, and a broad absorption band around 3262 cm^−1^ is attributed to O-H stretching [13,26,30,46]. The amide I and amide II bands did not change in the absence and presence of BE and/or GO, suggesting that the binding of anthocyanins and the formation of O/W emulsions had no impact on the secondary structure of hWPI.

The incorporation of 5% BE introduced characteristic phenolic signatures. The band at 1023 cm^−1^ indicates C–OH stretching in glycosylated phenols [47,48], confirming the presence of phenolic compounds and sugars typical of blueberry extracts. The band at 1080 cm^−1^ is associated with the aromatic CH bending and rocking and C-OH bending, while the band at 1152 cm^−1^ is related to the C-C-N stretching in amines [48]. The peak of 1743 cm^−1^ represents C=O stretching vibration in the carboxyl group (COOH), aldehyde group (CHO), or ester function (COOR). The peak of 2855 cm^−1^ is responsible for the stretching vibration of CH_2_ and CH_3_, and the peak of 2927 cm^−1^ is responsible for the asymmetric stretching vibration of C–H, indicating the presence of lipid alkyl chain [49]. The characteristic bands of BE or GO alone were similar to those of their mixed system, suggesting that there is no competition for their interaction with protein.

#### 3.1.4. Spatial Partition of Anthocyanin

In the aqueous dispersion of hWPI, anthocyanins may exist freely or be encapsulated by hWPI in the aqueous phase. The encapsulation efficiency of anthocyanins was about 62%, which was independent of the BE content (Table 1). In emulsions, polyphenols may also be adsorbed at the interface and transferred into the oil phase of emulsified oil droplets when added to the aqueous phase [20]. The total encapsulation of anthocyanins is the sum of anthocyanins in the emulsified droplets and encapsulated by hWPI in the aqueous phase. The percentages of free and totally encapsulated anthocyanin in GO emulsions were similar to those in the aqueous dispersion (Table 1), indicating that the oil addition did not affect the interaction between hWPI and anthocyanins. Anthocyanins are water-soluble polyphenols with limited affinity for lipid-rich substances [6]. It is thus speculated that anthocyanin molecules were mainly adsorbed by hWPI at the oil–water interface. The interfacial percentages of anthocyanins were approximately 36% and 42% when the GO content was 5% and 10%, respectively (Table 1). The increased partitioning of anthocyanins at the interface is due to the enhanced interface provided by GO. The anthocyanin percentage in the aqueous phase decreased as the GO content increased, which is consistent with the results of anthocyanins in rapeseed oil emulsions stabilized by whey protein [38]. The interfacial percentages of anthocyanins (Table 1) were greater than those of hWPI (Figure 2), supporting that the interaction with anthocyanins improved the interfacial activity of hWPI.

### 3.2. Characterization of Hydrogel and Emulsion Gel

#### 3.2.1. Visual Appearance

**Figure 4:**
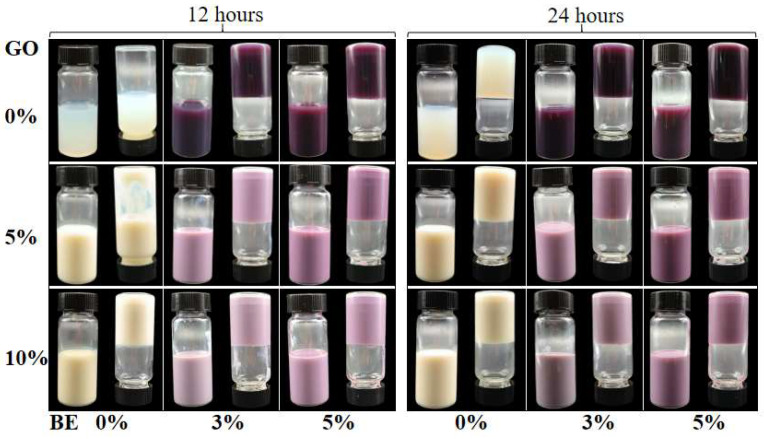
Appearance of cold-set WPI hydrogels and emulsion gels with various contents of blueberry extract (BE) and goji oil (GO) at 4 °C after 12 and 24 h. The concentration of calcium is 10 mM.

The solution of 6.5% hWPI remained in a liquid state at 4 °C after 12 h but formed a gel-like texture after 24 h, as evidenced by the lack of movement towards the bottom of the tubes (Figure 4). Hydrogels in the presence of 3% or 5% BE were already formed after 12 h, suggesting that BE enhanced the gelation of hWPI. The isoelectric point of whey protein is around 5.1 [20]. The pH values of the hWPI solutions in the presence of 0%, 3%, and 5% BEs were about 7.01, 6.85, and 6.73, respectively. It is thus speculated that the inclusion of BE enhanced the gelation of hWPI by reducing the net charges of whey protein and electrostatic repulsion among protein molecules. Moreover, anthocyanin-rich extracts strengthened the disulfide bonds and hydrophobic interactions within soy protein gels [13]. The emulsion gels formed 5% GO after 24 h and 10% GO after 12 h (Figure 4), suggesting that the oil droplets reinforced gel formation. The oil droplets acted as active fillers in the two-phase system and were distributed within the proteinaceous gel matrix, as described by Liang et al. [26]. The emulsion gels were cloudy compared to the hydrogels. For the emulsion gels containing 10% GO, all samples became opaque and remained at the top of the tubes when inverted after 12 h. The hydrogels and emulsion gels differed significantly in terms of their visual appearance and microstructure. The hydrogel primarily consists of a protein network stabilized by protein–protein interactions and interactions with blueberry extract [10]. On the other hand, the emulsion gel comprises a protein gel matrix with dispersed oil droplets, resulting in a more intricate microstructure [9].

#### 3.2.2. Morphological Characteristics

**Figure 5:**
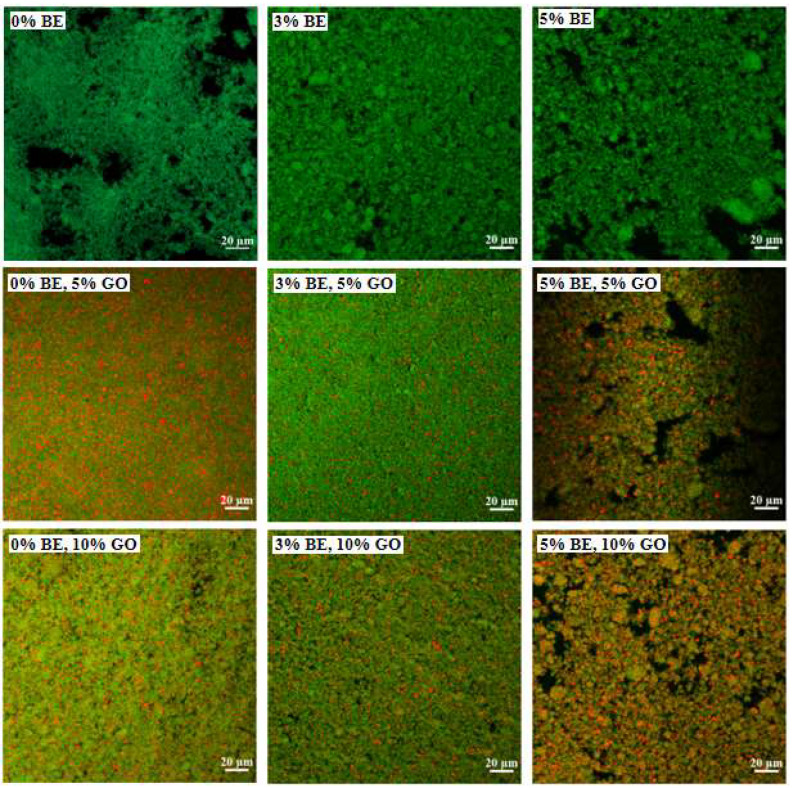
CLSM images of cold-set WPI hydrogels and emulsion gels with 0%, 3%, and 5% blueberry extract (BE) and with 0%, 5%, and 10% goji oil (GO).

Figure 5 illustrates CLSM images with green fluorescence for protein stained with Nile blue A and red fluorescence for GO stained with Nile red. The hydrogel of hWPI alone was a fine and dense network. The hydrogels became coarse with large aggregates upon the addition of BE. Aggregation was more pronounced with a visibly porous structure as the BE content increased to 5%. The opposite effect was reported for soy protein hydrogels prepared by heating 8% protein and 0–1.0% BE followed by acidification with GDL [13]. In emulsion gels, GO droplets were evenly dispersed throughout the network (Figure 5). The emulsion gels with 5% GO were more homogeneous and denser than the protein hydrogels, possibly due to the fact that the 5% GO emulsions had a more homogeneous size distribution than those of the hWPI solutions (Figure 1). However, the emulsion gels of 10% GO were heterogeneous with larger aggregates than those of 5% GO (Figure 5).

#### 3.2.3. Water-Holding Capacity

**Figure 6:**
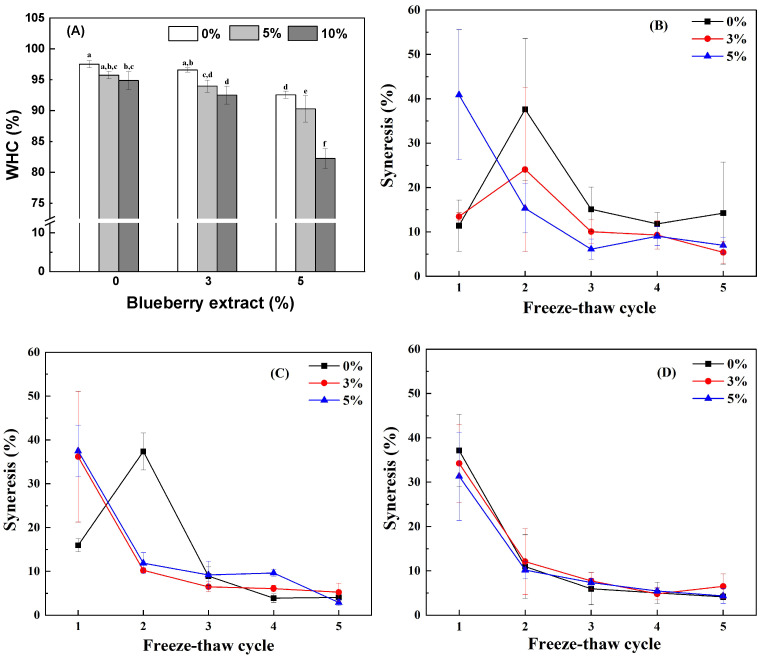
WHC (**A**) and syneresis (%) (**B**–**D**) of cold-set WPI hydrogels and emulsion gels with 0%, 3%, and 5% blueberry extract and with 0% (**B**), 5% (**C**), and 10% (**D**) goji oil. Values with different letters are statistically significantly different (*p* < 0.05).

Hydrogels and emulsion gels are functional materials that can incorporate different amounts of water into their networks [50]. Figure 6A depicts that the WHC of the WPI hydrogel was 98%. The WHC decreased as the BE content increased. Microstructure is an extremely important determinant of water retention when centrifugal force is exerted on the gel. The coarser the gel microstructure, the lower the WHC [51]. The increase in protein–protein interactions weakens the protein interaction with water, reducing the gel WHC [52].

Moreover, blueberry extract contains hydrophilic compounds such as anthocyanins [53], which may compete with gel matrix components for water-binding sites. This competition reduces the availability of water in the protein matrix, thereby limiting the gel’s ability to retain water. However, WHC decreased as the GO content increased (Figure 6A). The WHC of the emulsion gels with 5% BE and 10% GO was 82%. A higher oil content reduces the relative proportion of the aqueous phase, thereby limiting the water availability within the gel matrix. The physical volume occupied by oil droplets further restricts the space available for the water molecules. Additionally, the hydrophobic nature of oils displaces water and disrupts the structural integrity of the gel, thereby weakening its ability to hold water. This observation aligns with the findings of Lu et al. [54], who reported that increasing the olive oil content from 1% to 5% led to a decrease in WHC. However, this contrasts with a previous result that increasing the olive, soybean, and menhaden oil content from 0% to 20% resulted in a reduction in water loss in emulsion gels stabilized with egg-SPI proteins [55].

#### 3.2.4. Freeze–Thaw Stability

Freeze–thaw stability is crucial for evaluating the quality and durability of gel-based food products, as it reflects the product’s ability to endure repeated freezing and thawing cycles encountered during storage and transportation [56]. The syneresis (%) indicates the extent of water loss from the gel due to these cycles [57]. Figure 6B–D illustrate the syneresis (%) of the WPI hydrogels and emulsion gels across five freeze–thaw cycles. For WPI hydrogels with and without 3% BE and WPI emulsion gel with 5% GO and without BE, their syneresis was approximately 13% after the first freeze–thaw cycle, but this increased to 34% after the second cycle. Subsequently, the syneresis decreased with the number of freeze–thaw cycles. However, all other hydrogels and emulsion gels exhibited syneresis of about 36% after the first freeze–thaw cycle, which also decreased with subsequent cycles. It has been reported that freezing caused the aggregation of proteins and the coalescence of oil droplets, leading to a re-arrangement of the gel structure [58,59]. The high syneresis suggests a collapse of the gel structure following the freeze–thaw cycle. Therefore, comparatively, the WPI hydrogel with 3% BE and the WPI emulsion gel with 5% GO demonstrated high freeze–thaw stability. The decrease in syneresis with the number of freeze–thaw cycles may be attributed to the progressive disruption of the gel network due to ice crystal formation, leading to the development of large pores [26].

#### 3.2.5. Rheological Characteristics

**Figure 7:**
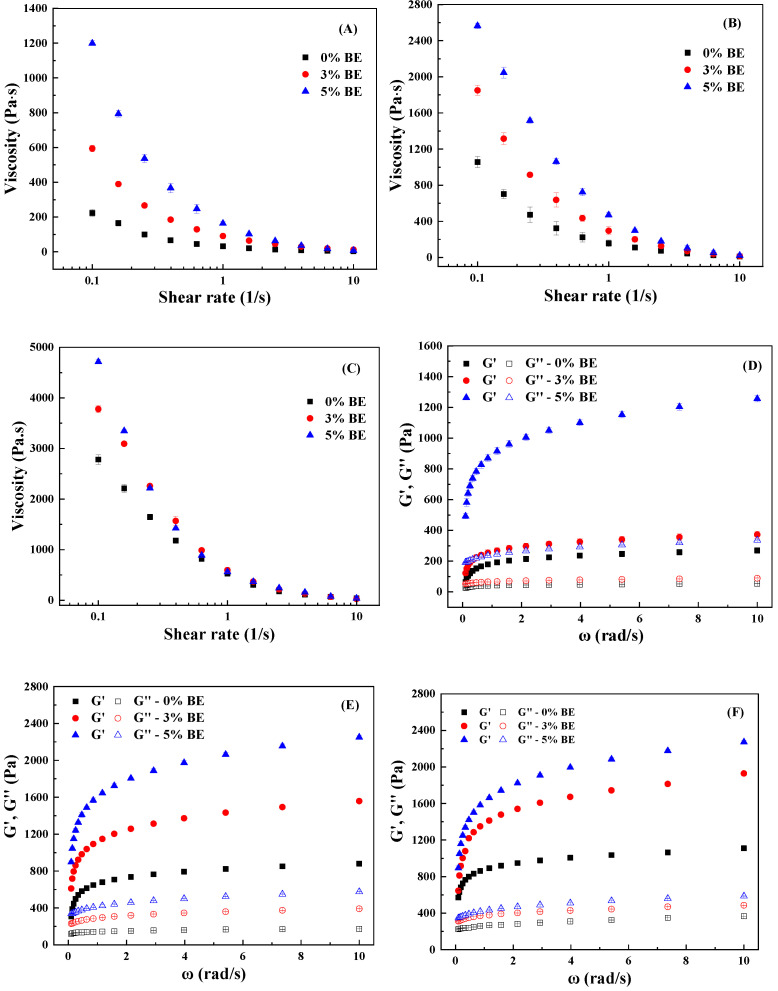
Viscosity (**A**–**C**) and G′ and G″ (**D**–**F**) of cold-set WPI gels and emulsion gels with 0%, 3%, and 5% blueberry extract (BE) and 0% (**A**,**D**), 5% (**B**,**E**), and 10% (**C**,**F**) goji oil.

The shear viscosity measurements involved large deformations that break down the gel network structure. Viscosity is an indicator of a substance’s internal friction between molecules or its capability to resist flow [60]. Figure 7A–C present that the viscosity of both hydrogels and emulsion gels decreased significantly with increasing shear rate, demonstrating shear-thinning behavior. The high shear rates partially disrupt the network structure of aggregated proteins, thereby reducing flow resistance and apparent viscosity [26]. Increasing the concentration of BE notably increased the viscosity of both hydrogels and emulsion gels at the same shear rate (Figure 7A–C). Additionally, the hydrogels exhibited lower apparent viscosity compared to the emulsion gels of the same composition, likely due to the presence of numerous aggregates or an open aggregate structure that traps more water (Figure 1 and Figure 6A).

The storage modulus (G′) quantifies the elastic or solid-like behavior of a material, reflecting the energy stored when the material is deformed. Conversely, the loss modulus (G″) measures the energy dissipated as heat, characterizing the viscous component of a material’s viscoelastic behavior [61]. In Figure 7D–F, G′ was higher than G″ in the overall frequency range, indicating that WPI hydrogels and emulsion gels exhibit a predominance of elastic behavior. Both G′ and G″ increased with the increasing content of BE, likely due to the strengthened network (Figure 5). The formation of covalent and non-covalent bonds between anthocyanins and WPI, particularly hydrogen bonds, hydrophobic interactions, and disulfide bonds, may promote cross-linking within the gel matrix [62]. The addition of GO significantly increased both G′ and G″, except that there was no change in G′ and G″ in the presence of 5% BE when the content of GO increased from 5% and 10% (Figure 7D–F). This effect is consistent with the change in the structure of the gel network (Figure 5). This enhancement is likely due to oil droplets acting as active filler particles that interact with the protein matrix, reinforcing the gel structure [26].

### 3.3. In Vitro Digestion of Hydrogels and Emulsion Gels

#### 3.3.1. Anthocyanin Release

**Figure 8:**
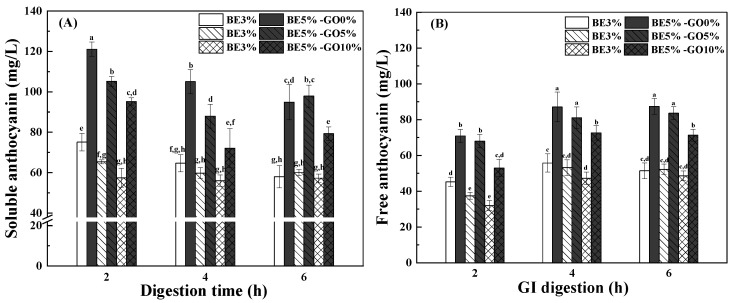
Soluble (**A**) and free anthocyanin (**B**) of WPI hydrogels and emulsion gels under simulated GI digestion (2 h gastric digestion followed by 4 h intestinal digestion). Values with different letters are statistically significantly different (*p* < 0.05). BE is the blueberry extract, while GO is goji oil.

Figure 8 presents soluble and free anthocyanin in simulated GI fluids. Anthocyanin as a flavylium cation is stable in the acidic condition [63]. After WPI hydrogels were digested in SGF for 2 h, the soluble anthocyanin was 75 and 121 mg/L when the BE was 3% and 5%, respectively (Figure 8A). The soluble anthocyanin decreased as the GO content increased, showing reductions of 57% and 95% at 10% oil content. A significant decrease in soluble anthocyanin occurred upon transfer to SIF, which can be explained by the pH-dependent chemical transformations of the anthocyanin structure. In neutral conditions, anthocyanins undergo a specific structural conversion where a nucleophilic attack by water on the flavylium cation leads to the formation of a hemiketal intermediate. This hemiketal can then undergo ring opening to first yield the cis-chalcone, followed by isomerization to form the more stable trans-chalcone, resulting in a colorless equilibrium mixture. This transformation process occurs through multiple reversible, pH-dependent chemical reactions, where the flavylium cation is stable in very acidic media while the trans-chalcone predominates in near-neutral conditions. The conversion involves sequential steps including hydration, ring opening, and isomerization, with the final trans-chalcone formation often taking several hours to reach equilibrium [6,7,14,64]. The dependence of the soluble anthocyanin on the BE and GO contents was similar in SGF and SIF, but the change became less pronounced with digestion time after 6 h. Figure 8B shows that the content of free anthocyanin was less than that of the soluble one in GI fluids. Soluble and free anthocyanin had a similar dependence on the BE and GO contents during simulated GI digestion (Figure 8). However, the contents of free anthocyanin in SIF were greater than those in SGF (Figure 8B). The contents of free anthocyanin were similar in SIF after 4 and 6 h. These results suggest that free and micelled anthocyanin co-existed in simulated GI fluids.

#### 3.3.2. Fatty Acid Composition Profile

**Figure 9:**
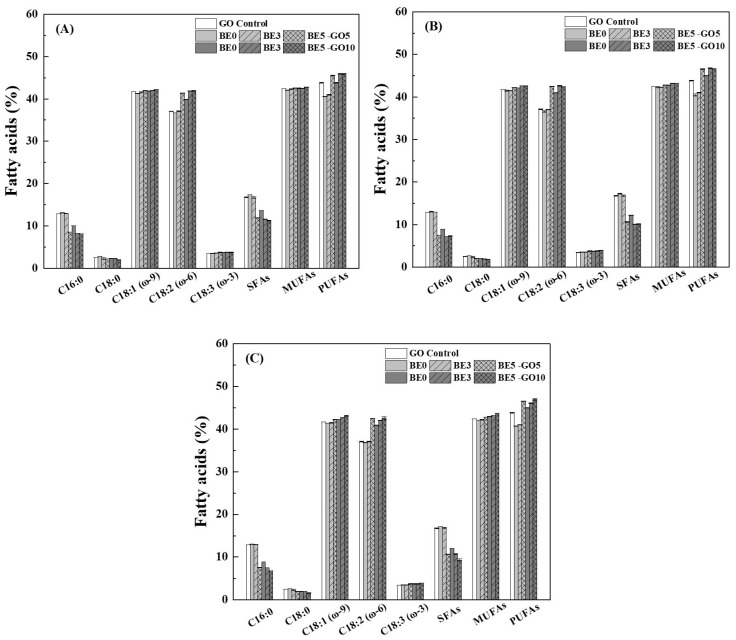
Fatty acids in the dissolution media of hydrogels and emulsion gels after 2 (**A**), 4 (**B**), and 6 h (**C**). BE for blueberry extract, GO for goji oil, SFAs for saturated fatty acids, MUFAs for monounsaturated fatty acids, and PUFAs for polyunsaturated fatty acids.

Table S2 shows the profile of fatty acids in GO. GO contains 16.76% saturated fatty acids (SFAs), 42.41% monounsaturated fatty acids (MUFAs), and 43.83% polyunsaturated fatty acids (PUFAs). Oleic acid (C18:1 ω-9) is predominant in MUFAs, while linoleic acid (C18:2 ω-6) is predominant, and linolenic acid (C18:3 ω-3) is minor in PUFAs [65]. The ω-6 and ω-3 PUFAs are essential since they are not synthesized by the body. GI digestion can change the FAs profile of food products [66]. Figure 9 shows the composition profiles of fatty acids in the dissolution media after digestion for 2, 4, and 6 h. In comparison with GO control, the percentage of PUFAs decreased to about 41% in the 5% GO gels but did not change in the 10% GO gels in SGF after 2 h (Figure 9A). The percentages of PUFAs were greater in the dissolution media of the 10% GO gels in SIF after 4 and 6 h than that of the 5% GO gels (Figure 9B,C), possibly due to the fact that the gel degradation was slower as the content increased by visual observation. The percentages of PUFA, especially linoleic acid, increased with the content of BE (Figure 9). The percentage of PUFAs was 46.98% in the dissolution media of the emulsion gels with 10% GO and 5% BE after 6 h (Figure 9C). These results suggest that the stability of PUFAs in the dissolution media was improved by the presence of BE, likely due to the antioxidant properties of anthocyanins. This is consistent with Zhou et al. [67], who found that fermented BE significantly reduced lipid oxidation and improved oxidative stability in emulsion-type sausage during storage. This effect was attributed to the potent antioxidant activity of anthocyanins, which protect PUFAs from oxidative degradation [53]. This is attributed to the greater stability of MUFAs compared to PUFAs in sensitive environments. The structural difference between these fatty acids makes MUFAs less susceptible to oxidative degradation, particularly under conditions involving heat or oxygen exposure. This finding is consistent with Grootveld et al. [68], who reported that MUFAs are more resistant to oxidation, whereas PUFAs tend to oxidize more rapidly in such conditions.

#### 3.3.3. Antioxidant Activity

**Figure 10:**
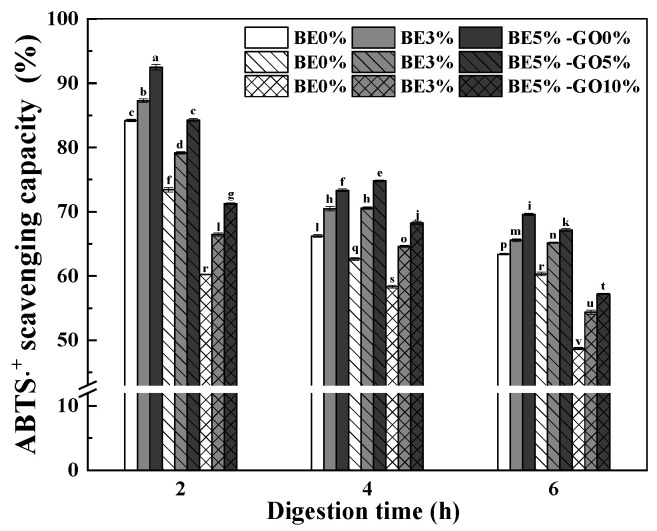
Antioxidant activity of WPI hydrogels and emulsion gels during simulated GI digestion (2 h in SGF followed by 4 h in SIF). Values with different letters are statistically significantly different (*p* < 0.05). BE is for blueberry extract, while GO is for goji oil.

Figure 10 illustrates the ABTS^∙+^ scavenging capacity of the dissolution media of hydrogels and emulsion gels. Whey proteins are hydrolyzed into peptides by pepsin and pancreatin, which contributes to the antioxidant activity [69] observed in the digested media of hydrogels and emulsion gels without BE. The antioxidant activity decreased from 84% in the hydrogel to 62% in the emulsion gel after 2 h of digestion and from 63% to 49% after 6 h of digestion with the increasing content of GO from 0% to 10% (Figure 10). Aboudzadeh et al. [70] found that higher oil content can reduce the effective concentration of antioxidants at the oil–water interface, where they are most needed to inhibit lipid oxidation. Since antioxidants distribute themselves between the oil, water, and interfacial regions in emulsions, their efficiency is directly dependent on their concentration at the interface. When the oil content increases, more antioxidants are solubilized in the oil phase, leaving fewer antioxidants at the interface to prevent oxidation, thus reducing overall antioxidant activity [70]. However, on the contrary, Zheng et al. [71] found that the emulsions with higher oil content displayed increased DPPH radical scavenging activity.

The antioxidant activity of BE is attributed to its high content of Cyd-3-O-GluCl, which is more efficient than other polyphenols such as catechins and flavonoids [72]. The ABTS^∙+^ scavenging capacity increased significantly (*p* < 0.05) with increasing BE content (Figure 10). For example, the scavenging capacity increased from 84% to 92% for hydrogels after 2 h of digestion and from 48% to 57% for 5% BE and 10% GO emulsion gels after 6 h of digestion as the BE content increased from 0% to 5%. This positive correlation is attributed to the hydrophilic nature of anthocyanin, which enabled the polyphenol molecules to diffuse readily into hydrophilic media [73]. A significant decrease in the scavenging capacity was observed as digestion progresses from the gastric to the intestinal phase (Figure 10) due to the degradation of anthocyanin and unsaturated fatty acids (Figure 8 and Figure 9). These results are consistent with the report of Xu et al. [74], in that the antioxidant activity of the double emulsions containing anthocyanins was higher in simulated gastric digestion compared to intestinal digestion in terms of DPPH, ORAC, and FRAP assays.

## 4. Conclusions

The inclusion of anthocyanins-rich blueberry extract (BE) caused the aggregation of hWPI particles but improved the protein interfacial adsorption and reduced the size distribution of goji oil (GO) emulsions. The formation of goji oil emulsions had no influence on the encapsulation efficiency of anthocyanins by WPI. The gelation time of hWPI particles and emulsions was shortened to form a dense network with decreased water-holding capacity by the inclusion of BE and goji oil. Anthocyanins-rich BE was used as a model of bioactive compounds as well as the adjuvant of calcium-induced gelation. The network became more homogeneous and denser in the presence of 3% blueberry extract and 5% goji oil. The WPI hydrogel with 0% and 3% blueberry extract and WPI emulsion gel with 5% goji oil had high freeze–thaw stability. The co-inclusion of blueberry extract and goji oil increased the syneresis rate during the freeze–thaw cycle. All WPI hydrogels and emulsion gels exhibited predominantly elastic behavior with improved network strength when increasing the BE and GO contents. During in vitro digestion, soluble and free anthocyanins reduced with the GO content but increased with the BE content. Free anthocyanins were greater in simulated intestinal fluid than in simulated gastric fluid, but soluble anthocyanins were contrary. The digested stability of polyunsaturated fatty acids (PUFAs) was improved by blueberry extract. The antioxidant activity of the digested medium increased with the BE and GO contents. Future works will be performed to investigate the effect of BE and GO co-encapsulation on health benefits. This work contributes valuable knowledge to the effect of bioactive compounds on the formation of gel-based carrier systems and the effect of the carrier systems on the co-delivery of bioactive compounds, providing a potential application for the development of health foods with multiple nutrition properties.

## Figures and Tables

**Table 1:** Spatial partition of anthocyanin (AC) in heat-denatured WPI suspensions and O/W emulsions.

GO (%)	BE (%)	Aqueous AC (%)	Interfacial AC (%)	Free AC (%)	hWPI-Encapsulated AC (%)	Totally Encapsulated AC (%)
0	3	100.0 ± 0.0 ^a^	-	35.4 ± 9.6 ^a^	64.6 ± 9.6 ^a^	64.6 ± 9.6 ^a^
	5	100.0 ± 0.0 ^a^	-	40.7 ± 8.1 ^a^	59.3 ± 8.1 ^a^	59.3 ± 8.1 ^a^
5	3	62.0 ± 1.2 ^c^	38.0 ± 1.2 ^b^	31.0 ± 6.6 ^a^	31.06 ± 6.0 ^b^	69.0 ± 6.6 ^a^
	5	65.8 ± 1.8 ^b^	34.2 ± 1.8 ^c^	37.6 ± 9.2 ^a^	28.2 ± 7.8 ^b^	62.4 ± 9.2 ^a^
10	3	56.5 ± 2.3 ^d^	43.5 ± 2.3 ^a^	29.9 ± 3.3 ^a^	26.6 ± 1.0 ^b^	70.1 ± 3.3 ^a^
	5	58.8 ± 1.5 ^d^	41.2 ± 1.5 ^a^	32.6 ± 9.2 ^a^	26.2 ± 7.7 ^b^	67.4 ± 9.2 ^a^

All results are expressed as the means ± standard deviation; n = 3; means followed by different superscript letters differ significantly (*p* < 0.05). GO is goji oil, while BE is blueberry extract.

## Data Availability

Data are contained within the article and Supplementary Materials.

## Supplementary Materials

The following supporting information can be downloaded at: https://www.mdpi.com/article/10.3390/antiox14010060/s1, Figure S1: Elution curves of cyanidin-3-O-glucoside, (A) full wavelength vs. elution time, (B) the absorbance at 520 nm vs. elution time, (C) the absorption spectrum at the elution time of cyanidin-3-O-glucoside. Figure S2: Elution curves of blueberry extract, (A) full wavelength vs. elution time, (B) the absorbance at 520 nm vs. elution time, (C) the absorption spectrum at the elution time of cyanidin-3-O-glucoside. Table S1: pH of heat-denatured WPI suspensions and O/W emulsions. Table S2: Profile of fatty acids in goji oil.
