# Supplementary material for: Whey Protein Hydrogels and Emulsion Gels with Anthocyanins and/or Goji Oil: Formation, Characterization and In Vitro Digestion Behavior
Source: Antioxidants (Basel). 2025 Jan 7;14(1):60. doi: 10.3390/antiox14010060 (PMC11760487; doi:10.3390/antiox14010060)
Supplement: Supplementary file 1 [file antioxidants-14-00060-s001.zip › antioxidants-3362339-supplementary.pdf]

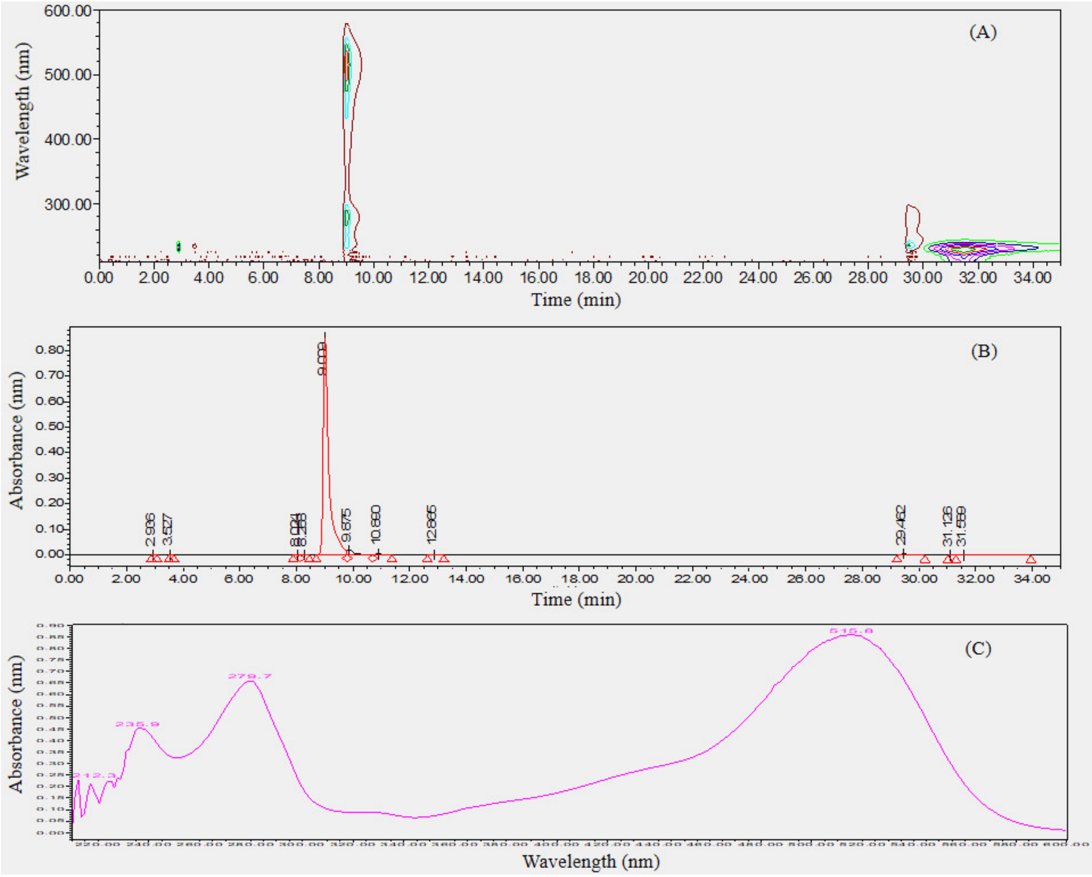

Figure S1 Elution curves of cyanidin-3-O-glucoside, (A) Full wavelength vs elution time, (B) The absorbance at 520 nm vs elution time, (C) The absorption spectrum at the elution time of cyanidin-3-O-glucoside.

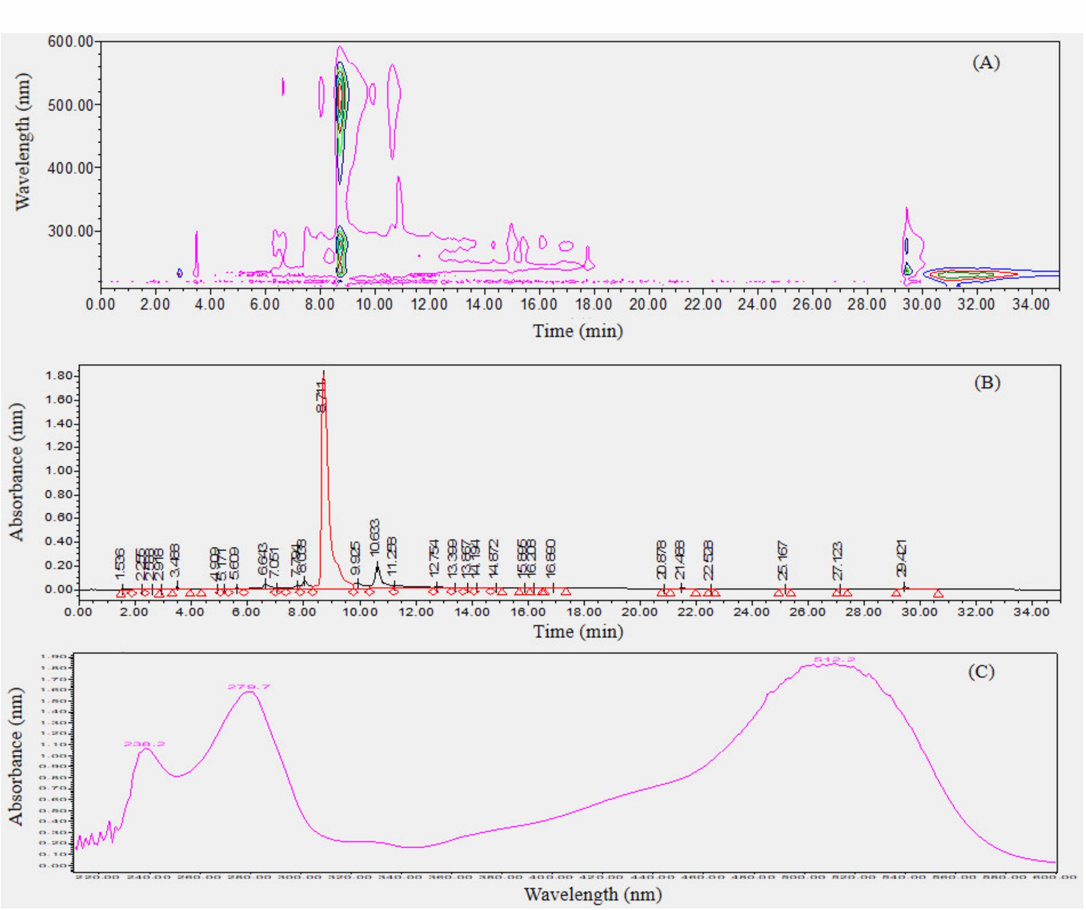

Figure S2 Elution curves of blueberry extract, (A) Full wavelength vs elution time, (B) The absorbance at 520 nm vs elution time, (C) The absorption spectrum at the elution time of cyanidin-3-O-glucoside.

Table S1 pH of heat-denatured WPI suspensions and O/W emulsions.

Blueberry extract (%)	Goji oil (%)		
	0	5	10
0	7.01	7.00	7.01
3	6.80	6.92	6.82
5	6.72	6.72	6.74

Table S2 Profile of fatty acids in goji oil

Fatty acids		Percentage (g/100 g oil)
Myristic acid	C14:0	0.21 ± 0.00
Pentadecanoic acid	C15:0	0.01 ± 0.01
Palmitic acid	C16:0	12.82 ± 0.01
Palmitoleic acid	C16:1 (ω-7)	0.17 ± 0.00
Margaric acid	C17:0	0.00 ± 0.00
Heptadecenoic acid	C17:1 (ω-7)	0.00 ± 0.00
Stearic acid	C18:0	2.53 ± 0.02
Oleic acid	C18:1 (ω-9)	41.68 ± 0.01
Linoleic acid	C18:2 (ω-6)	37.07 ± 0.09
Linolenic acid	C18:3 (ω-6)	0.33 ± 0.00
Linolenic acid	C18:3 (ω-3)	3.43 ± 0.01
Arachidic	C20:0	0.59 ± 0.01
Eicosenoic acid	C20:1 (ω-11)	0.56 ± 0.01
Behenic acid	C22:0	0.31 ± 0.04
Lignoceric acid	C24:0	0.29 ± 0.01
Saturated fatty acids		16.76 ± 0.08
Monounsaturated fatty acids		42.41 ± 0.01
Polyunsaturated fatty acids		43.83 ± 0.08

All results are expressed as the means ± standard deviation, n = 3.
